# Chiral 480 nm absorption in the hemoglycin space polymer: a possible link to replication

**DOI:** 10.1038/s41598-022-21043-4

**Published:** 2022-09-28

**Authors:** Julie E. M. McGeoch, Malcolm W. McGeoch

**Affiliations:** 1 High Energy Physics DIV, Smithsonian Astrophysical Observatory, Center for Astrophysics, Harvard & Smithsonian, 60 Garden St, MS 70, Cambridge, MA 02138 USA; 2 PLEX Corporation, 275 Martine St, Suite 100, Fall River, MA 02723 USA

**Keywords:** Biophysics, Astronomy and planetary science

## Abstract

A 1494 Dalton hemoglycin space polymer of Glycine_18_ Hydroxy-glycine_4_ Fe_2_O_4_ termed the “core unit” is part of a polymer of Glycine, Si, Fe and O that forms tubes, vesicles and a lattice structure. It has been isolated from four different CV3 meteorites and characterized by mass spectrometry, FIB/SIMS and X-ray analysis. In quantum calculations (HF and DF wB97X-D 6-31G) the polymer has an absorption at 480 nm that is dependent on rectus “R” (= dextro D) chirality in a hydroxy glycine residue whose C-terminus is bonded to an iron atom. The absorption originates in the Fe II state as a consequence of chiral symmetry breaking. In confirmation of theory, measurements at Diamond Light Source UK, on crystals of hemoglycin derived from Acfer-086 and Sutter’s Mill meteorites have shown a strong 483 ± 3 nm absorption that confirms the proposed location of hydroxy glycine residues within the polymer. A high 483 nm to 580 nm absorption ratio points to an “R” chirality excess in hemoglycin, suggesting that 480 nm photons could have provided the energy for its replication in the protoplanetary disc.

## Introduction

Hemoglycin was shown theoretically^1^ to absorb at 480 nm provided its structure had “R” chiral hydroxyglycine near a C-terminus bond to iron. We now add experimental absorption data that confirms the theory. This light-based molecular data means that in principle astronomers can detect hemoglycin in protoplanetary discs. Chiral asymmetry became an element in the present work when our measured 480 nm to 580 nm absorbance ratio exceeded the calculated ratio for a racemic mix of hemoglycin molecules with equal “R” and “S” abundances. The background to chiral asymmetry in meteoritic material is briefly reviewed before we return to the absorbance calculations and experimental results.

In terrestrial biology, the dominance of “S” (L) over “R” (D) amino acids, first noted by Louis Pasteur^2^, has inspired much work on meteorites, the presumed source of much of Earth’s organic matter^3^, to find whether “S” chirality preceded life on Earth, and what its source could be. Using the elevated ^15^N signature of meteoritic amino acids as a guarantee of extra-terrestrial origin, Engels and Macko^4^ reported D/L ratios of 0.5 and 0.3 respectively for amino acids alanine and glutamic acid in Murchison, an indication that there could have been a pre-biotic influence on chirality, as opposed to the un-biased (racemic) expectation of D/L = 1. Results on non-biological *α*-methyl amino acids in Murchison^5^ and in Murchison, Orgueil and four other meteorites confirmed this finding^6^. It was noted that there had not been any report of a D/L ratio greater than 1, which suggested to the authors of^6^ that inside a meteorite parent body an initially racemic chemical stock was entering a chirally-biased synthesis to produce L dominance in certain amino acids.

However, insoluble organic material (IOM) which comprises much the greater part of meteoritic organics relative to the soluble amino acids, showed a definite “R” (D) bias in extracts from Allende, Murray and Murchison^7^, when tested via the Soai reaction^8^. This method initiates autocatalytic amplification of the chirality when a certain molecule is added to the sample. When the sample is non-chiral the result is growth of the added molecule equally to either “S” or “R” dominance. However, in the IOM of these meteorites there appeared a consistent “R” signature due to a source that was not molecularly identified. Hemoglycin has IOM characteristics including its relative insolubility in water, its nitrogen content that is a feature of the IOM^9^ and its very high isotope enrichment^10^, hence the present finding of “R” excess may be connected directly, or indirectly, with the findings of^7^.

The structural meteoritic polymers discovered and characterized in our prior work^10–14^ are here modeled at a high level of quantum chemistry to predict their optical absorptions for telescopic studies of protoplanetary disc evolution. Being widespread in carbonaceous chondritic meteorites of the early CV3 type and having ^2^H and ^15^N isotope levels characteristic of comets, these molecules could have played a role in the accretion of our solar system and therefore might be observable in other circumstellar discs. The molecular structure of these space polymers, that we term in general hemoglycin, contains iron atoms that close the ends of anti-parallel polyglycine chains in a previously unknown iron configuration.

**Figure 1:**
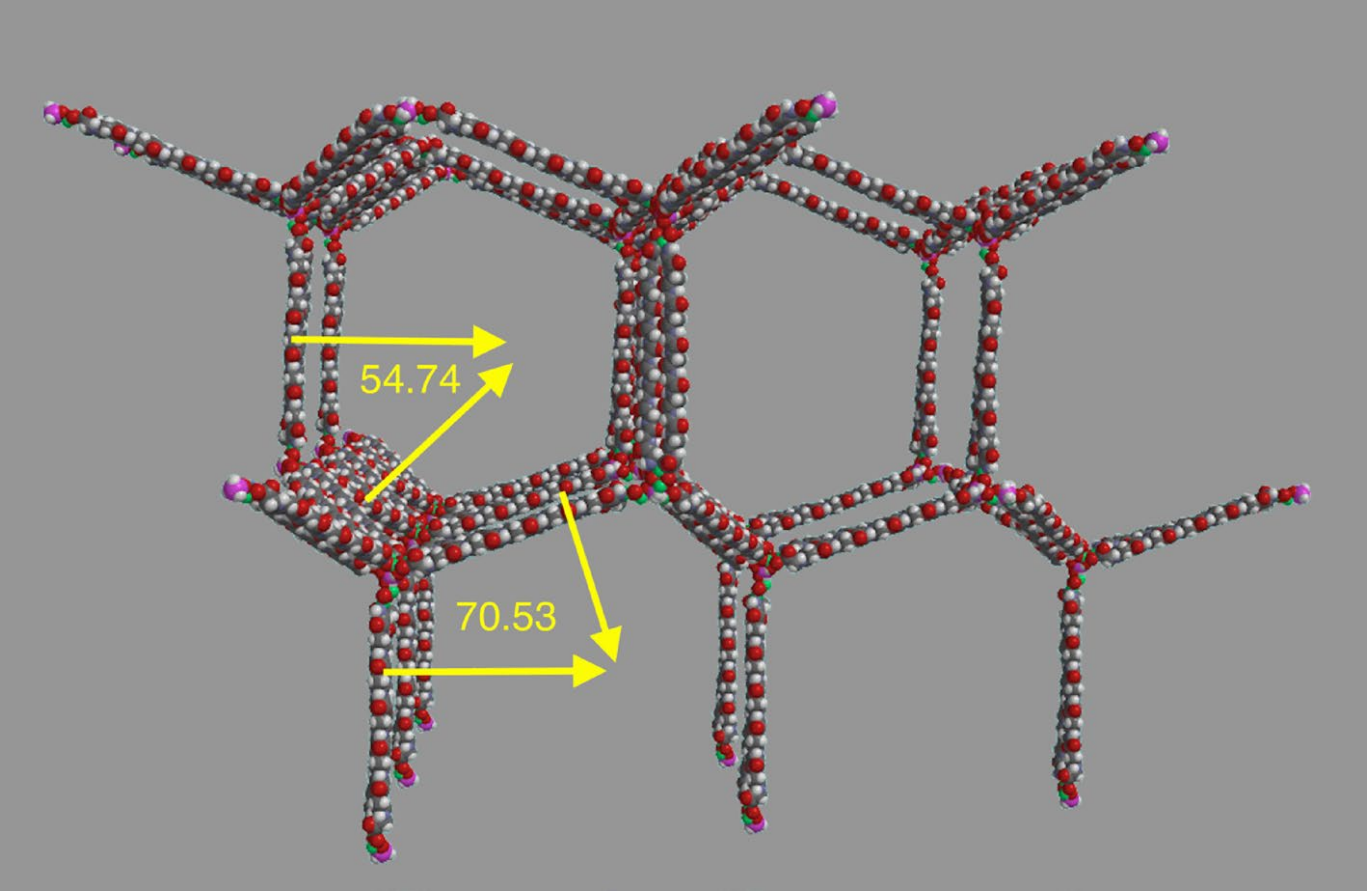
Lattice form of the polymer^14^ built of modified core units, here at 1638 Da, linked by silicon atoms. The tetrahedral angles have been confirmed in X-ray scattering. Space filling model. Atoms: hydrogen white, carbon black, nitrogen blue, oxygen red, iron green, silicon pink. Spartan '20 Version 1.1.5 (220607) (Mac OS 12.5.1).

The 1494 Da core unit (Glycine_18_ Hydroxy-glycine_4_ Fe_2_O_4_) and its associated polymers have been determined by extracting the molecule from micron particles of CV3 meteorite samples and then analyzing the extracts by mass spectrometry^10–13^ and X-ray diffraction^14^ with supporting measurements of ^15^N^15^ via FIB/SIMS. The MALDI mass spectrometry technique basically provides data on an intact molecule and/or its fragments with data displayed as peak amplitude versus mass to charge ratio (*m/z*). Thousands of MALDI peaks have been analyzed^10–13^ allowing models of the polymer structure to be built by software, aided by X -ray diffraction data of crystals of the polymer^14^. The major m/z peaks had upon analysis a universal core unit of mass 1494 Da, which would not have been expected following a random polymerization, and therefore strongly suggested that template replication had been operative within our protoplanetary disc from which the asteroids formed. This polymer forms rolled-up tubes, can cover a surface forming spherical vesicles^10^, and self-organizes into a three-dimensional low-density lattice^14^ that is depicted in Fig. 1. The source meteorite samples were Allende^11,12,15^, Acfer-086^10–12,14,15^ and Kaba^10,14^ while Sutter’s Mill crystals in the present work showed X-ray diffraction and UV/visible absorption similar to those in Acfer 086.

**Figure 2:**
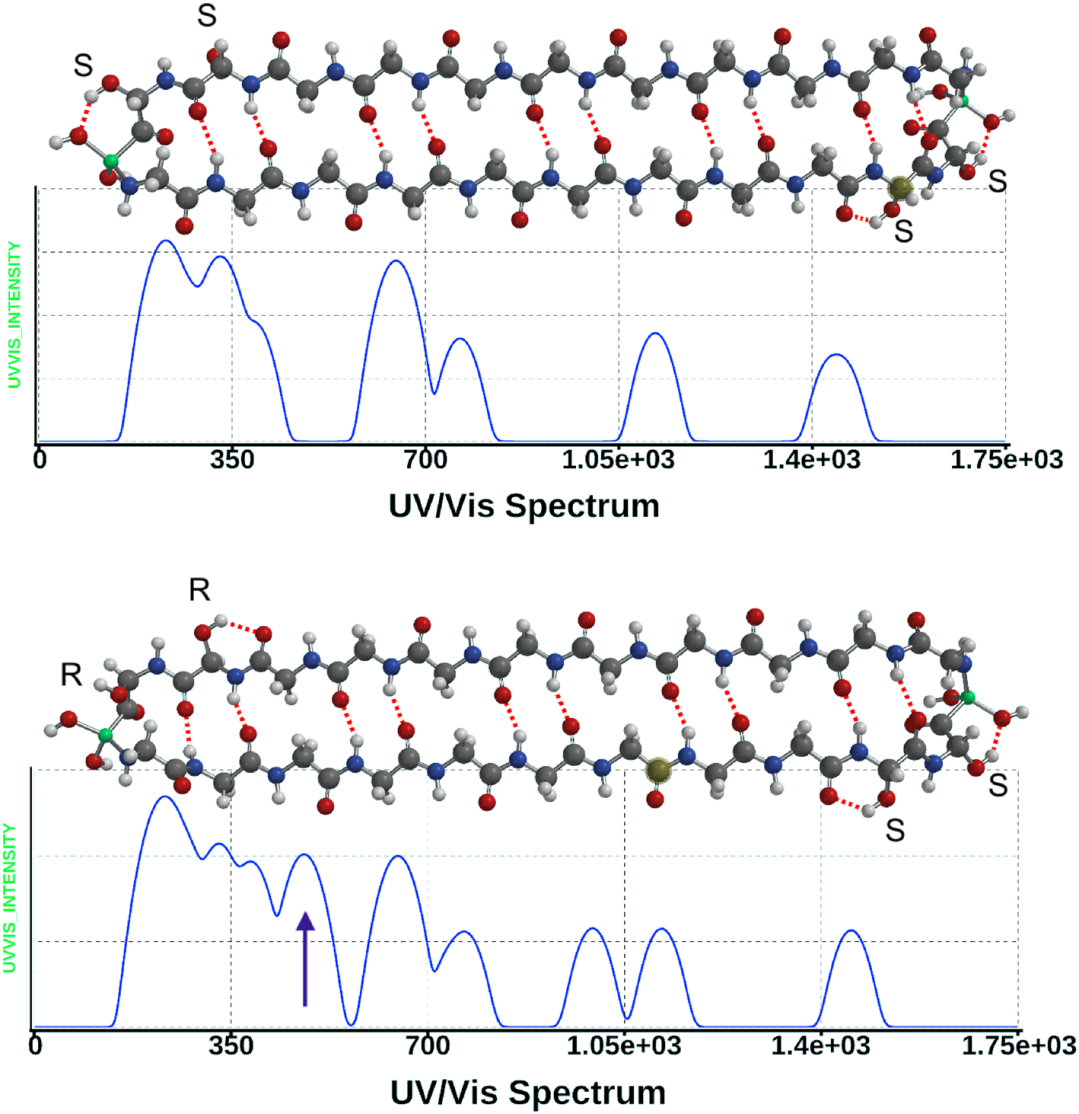
(**a**) (Top) UV/visible/IR spectra from the core polymer where the 4 hydroxy-glycine units all have ‘S’ (sinister/laevo S/L) chirality. There is no absorption at 480 nm. Graph axes: Vertical; calculated transition strengths on a log_10_ scale: Horizontal; wavelength (nm). The molecular model format is ball and spoke. Atom labels: hydrogen white, carbon black, nitrogen blue, oxygen red, iron green. (**b**) (Bottom) spectrum from the core polymer with an ‘R’ chirality hydroxy-glycine adjacent to an Fe atom at the peptide C-terminal. Spartan '20 Version 1.1.5 (220607) (Mac OS 12.5.1).

UV/visible and IR calculations were run on the core protein and on pared down versions of that structure using equilibrium geometry, Hartree–Fock, 3-21G. Shorter versions could be used to reduce computational time because the length of the glycine chain did not affect the absorptions which explicitly were associated with the terminal iron atoms and their immediate molecular surroundings. Absorption wavelengths and strengths were calculated to 20 excited states, which covered the range down to the mid-ultraviolet. Transition strengths were typically plotted on a log_10_ vertical scale unless mentioned otherwise, and an artificial width of 40 nm was applied to the spectral peaks to simulate the typical molecular broadening via vibrations (Fig. 2). The calculations were all in gas phase, which we believe is more appropriate for molecules in molecular clouds or discs. The pared down structures had less glycine units per anti-parallel beta sheet chain and beyond the 2Fe case included a core with only a single Fe, loops of glycine with no Fe, the core minus OH groups, different placements of the OH groups and changes to the chirality of the OH groups.

Following the here-reported discovery via modeling of a prominent 480 nm absorption only predicted to occur when an “R” chirality hydroxy glycine residue was C-terminal connected to an iron atom, crystals were probed for absorption between 300 and 840 nm on the tunable Microfocus MX I24, beamline at the Diamond Light Source synchrotron UK. All of the calculated UV/visible absorptions in this range were observed in hemoglycin crystals identified by X-ray diffraction at both APS and Diamond synchrotrons, confirming the present molecular model. Furthermore, the excess strength of 480 nm over 580 nm features pointed to a substantial “R” chirality excess. Either this was generated by what appears to be an unlikely bias in the hydroxylation, or it was generated by template replication driven toward “R” chirality because it depended upon the energy of 480 nm photons only absorbed by that chirality. Modeling (S3) showed that the latter mechanism was feasible as an explanation of the observed “R” excess, supporting the likely existence of replication.

## Results

The “core unit”, comprises two poly-glycine chains of length 11 residues in an anti-parallel beta sheet with iron atoms joining the chains at each end^10^, illustrated in Fig. 2. Four of these glycine residues have OH groups on the alpha carbon units, in ‘S’ or ‘R’ chirality (Laevo- or Dextro- rotatory, respectively). An absorption at 480 nm is induced only when there is an ‘R’ chirality hydroxy-glycine connected via its peptide C-terminal to a terminal iron atom. The absorption is absent in all other cases involving (plain) glycine or ‘S’ hydroxy-glycine at either the C-terminal or the N-terminal adjacent to an Fe atom, also it is absent when ‘R’ hydroxy-glycine has its N-terminal next to iron. The origin of this induced optical transition lies in the symmetry-breaking that occurs when an otherwise symmetrical iron atom is attached via covalent bonds to an asymmetric polypeptide structure with directionality, i.e. the iron orbitals are constrained from rotating relative to the polypeptide structure, which itself has chirality. We find that the absorption relates to the Fe II (Fe^+^) state of iron in specific molecular surroundings.

Mass spectrometry had revealed that an even number of glycine residues was contained in the dominant m/z peaks, suggesting that there could be a pairing of equal length single strands of poly-glycine. Energy minimization pointed to an anti-parallel pairing owing to the strength of the {C=O:::H-N} hydrogen bonds between strands in that orientation. This structure, referred to as poly-glycine I, is fully described by Lotz^16^ and Moore and Krimm^17^ in their analyses of its infrared (IR) spectrum vibrations. Here, each “core unit” chain has 11 glycine residues with two of these modified into hydroxy-glycine (Gly_OH_). The chains are linked at the ends via Fe atoms. Two possible versions of the core unit, differing by chirality, are shown in Fig. 2 in equilibrium geometry. Our earlier mass spectrometry work indicated via study of fragments that the hydroxy-glycine residues were very probably adjacent to terminal Fe atoms^10^, but mass spectrometry alone could not determine residue chirality.

One example of chiral induction of a 480 nm absorption is shown in Fig. 2 in which the vertical scale represents calculated transition strengths on a log_10_ scale and the chirality of Gly_OH_ is indicated by ‘S’ or ‘R’. The range of calculations covered 5, 7, 9, 11 and 13 residue chains, with two, one or zero Fe atoms. When Fe was absent the polymer was given an additional peptide bond at that location, to make a continuous loop. The cases without any Fe had only a main deep ultraviolet absorption in the region of 150–200 nm (S1 Fig. 1) and did not exhibit any of the visible or near infrared absorptions discussed here. The latter were associated only with the presence of iron, and furthermore with single iron atoms that could be identified by the Mulliken charge as being at one or the other end of the molecule. To complete calculations on the nine cases that covered zero, ‘S’-chiral and ‘R’-chiral Gly_OH_ located on either the C-terminus or the N-terminus adjacent to only one Fe atom, we reduced the chain length to 7 residues, having first determined that the results did not depend upon chain length from 7 thru 13, but only upon the local arrangement around one iron atom. The calculated absorption wavelengths for these 9 cases are listed in Table 1.

**Table 1:** Calculated absorption wavelengths for the “7-residue chain” with zero, one or two Gly_OH_ residues at one end only, coded by its absence (0) or location on {N-terminal, C-terminal} and chirality (S or R) via {(0,S,R),(0,S,R)}.

State indice	Case 1 {0,0}	2 {0,S}	3 {0,R}	4 {S,0}	5 {S,S}	6 {S,R}	7 {R,0}	8 {R,S}	9 {R,R}
1	1221	1408	1598	1252	1457	1510	1406	1827	1753
2	1221	1220	1221	1221	1221	1221	1216	1220	1222
3	1045	1120	1044	1045	1044	1044	1041	1080	1058
4	1044	1045	1028	959	1041	998	1008	1044	1045
5	757	764	757	816	827	757	762	766	757
6	757	757	659	757	757	696	753	757	726
7	596	639	596	596	597	596	596	596	596
8	596	596	**482**	556	595	**475**	530	554	**475**
9	361	388	398	365	394	396	370	402	409
10	361	361	380	361	361	380	362	361	397
11	347	347	361	347	347	361	361	346	361
12	346	335	347	346	332	347	347	346	347
13	331	332	332	332	330	331	333	331	332
14	331	326	330	332	322	330	331	316	327
15	291	319	291	293	319	291	292	311	291
16	291	291	274	291	291	263	282	291	276
17	254	269	254	254	264	254	254	254	254
18	254	254	240	250	254	236	243	253	243
19	226	230	227	226	226	226	226	227	233
20	226	226	226	218	224	223	223	226	226

For example, {0,R} refers to a C-terminal R Gly_OH_ only, with unmodified glycine at the N-terminal. Wavelengths (nm) and transition strengths are from the RPA calculations.

Significant values are given in bold.

Without Gly_OH_, in column 1 of Table 1, the calculated absorptions rise in pairs, which correlate with first one, then the other iron atom. Generally, the lowest energy absorption varies toward longer wavelength with any of the Gly_OH_ residues present (first row of Table 1). The second row relates to the distal iron atom, and hence is immune to environmental changes around the first Fe where hydroxylations are located.

### Interpretation of the calculated spectra relative to Fe structure

**Figure 3:**
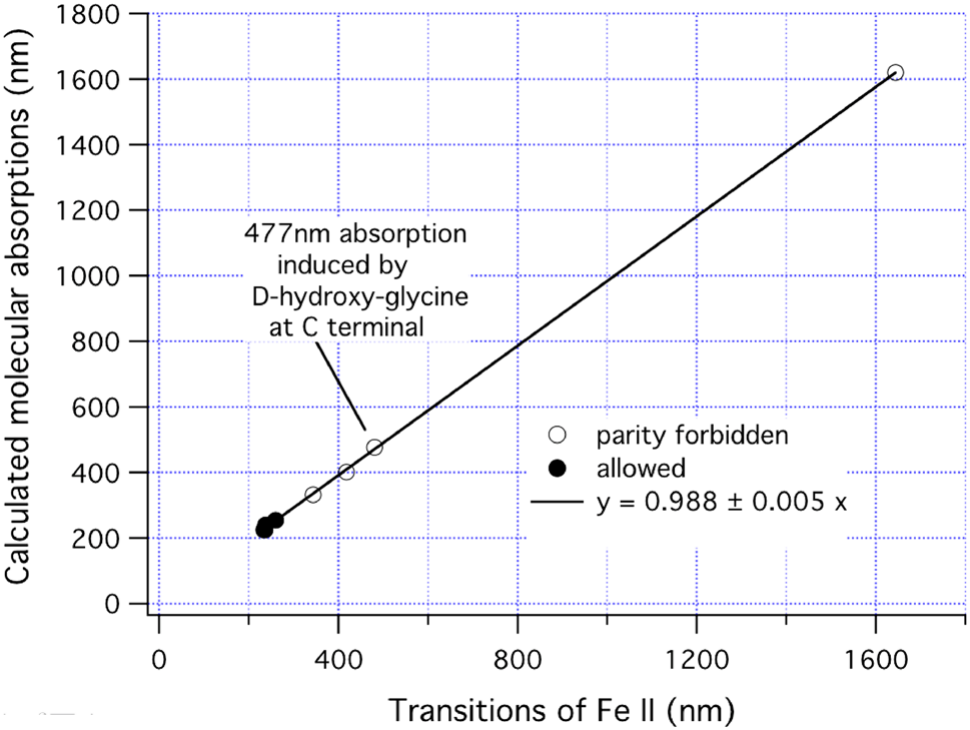
Correlation between transitions in Fe II (Fe^+^) and RPA calculated transitions for molecular cases with R(D) chirality hydroxy-glycine in C-terminal contact with Fe.

In cases 3, 6 and 9 of Table 1, which have an R-Gly_OH_ C-terminal bonded to Fe, we noted that there was a good correlation between the calculated absorption wavelengths (in the random phase approximation, RPA) and a set of transitions in the Fe II (Fe^+^) spectrum. The wavelengths of corresponding states in columns 3, 6 and 9 were averaged and plotted in Fig. 3 against the Fe^+^ (Fe II) transitions^18–20^ listed in Table 2. An exact linear correlation was seen, within which the open circles represented transitions of Fe II that were not allowed due to parity, while the solid circles showed allowed transitions. In Fe II there are two close-lying ground states, the Fe II a^4^F_9/2_ quasi-ground state lying only 1872 cm^−1^ above the true ground state a^6^D_9/2_. Many of the calculated molecular transition wavelengths of Table 1 could be traced to an atomic “root” in either the a^4^F_9/2_ or the a^6^D_9/2_ state of Fe II as listed in Table 2. In confirmation of the Fe charge state, all the ground states listed in Table 2 had a Mulliken charge of 1.06 units, effectively acting as Fe^+^ (Fe II) ions. The calculated strength of the 477.5 nm absorption was 0.0116, averaged over cases 3, 6 and 9, which greatly exceeded the strengths of the other parity-forbidden transitions of Table 2, indicating a moderate strength for the induced absorption at 477.5 nm. Following absorption, re-radiation from this transition would be expected to have a decay life of about 300 ns in the absence of further energy transfer into vibrations of the molecule.

**Table 2:** List of Fe II transitions either forbidden due to parity (*parity X*) or fully allowed, compared to RPA results for averaged cases 3, 6 and 9 of Table 1 involving R(D) hydroxy-glycine residues with C-termini on Fe.

Term *g.s.*→*upper*	Energy (cm^−1^)	Wavelength (nm)	Strength	RPA result (nm)	RPA strength
$a^{6} D_{9/2} \to z^{6} D_{9/2}^{o}$	38,458	260	Allowed	254	0.0012
$a^{6} D_{9/2} \to z^{6} F_{11/2}^{o}$	41,968	238	Allowed	240	0.141
$a^{6} D_{9/2} \to z^{6} P_{7/2}^{o}$	42,658	234	Allowed	225	0.041
$a^{4} F_{9/2} \to {}^{4}D_{7/2}$	6083	1644	Parity X	1620	0.00001
$a^{4} F_{9/2} \to b^{4} F_{9/2}$	20,765	481	Parity X	477.5	0.0116
$a^{4} F_{9/2} \to a^{4} G_{11/2}$	23,933	418	Parity X	401	0.00040
$a^{4} F_{9/2} \to b^{4} D_{7/2}$	29,053	344	Parity X	332	0.0071
$a^{4} F_{9/2} \to z^{4} F_{9/2}^{o}$	42,360	236	Allowed	228	0.062

An offset of 2050 ± 460 cm^−1^ in energy was seen between CIS and RPA levels of calculation, with the CIS energies being higher. We identified this constant energy difference with the 1872 cm^−1^ difference between the two Fe II ground states, indicating a slight difference of outcome between the two methods. As we were able to correlate the calculated RPA results quite accurately with excitations from the Fe II a^4^F_9/2_ quasi-ground state (Table 2) we believe that the RPA results quoted throughout this paper are probably reliable to within a few tens of nm.

### Infrared spectra

**Figure 4:**
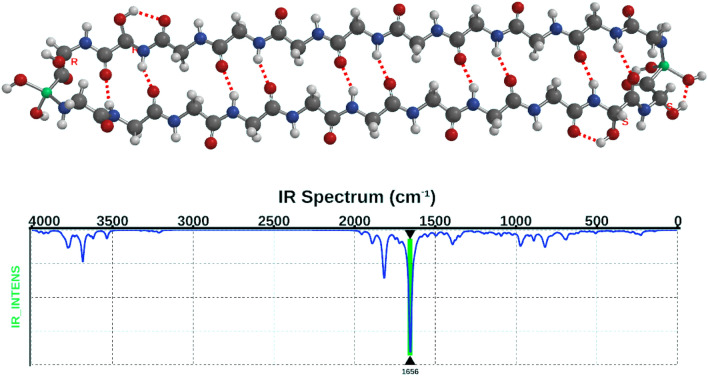
IR absorption from the core of Glycine_18_ Hydroxy-glycine_4_ Fe_2_O_4_. An absorption peak around 1651–8 cm^−1^ (6.0 µm), shown here at 1656 cm^−1^ is the typical “amide I” absorption for the backbone of the anti-parallel beta sheet^17^. This IR spectrum is typical of the core and of the pared down conformers. Molecule format is ball and spoke. Atom labels: hydrogen white, carbon black, nitrogen blue, oxygen red, iron green. Spartan '20 Version 1.1.5 (220607) (Mac OS 12.5.1).

We calculated the infrared spectra as a guide to astronomical observations of circumstellar discs that could contain space polymers. The infrared (IR) spectra have no major vibration specific to the R/D chirality or positioning of the hydroxy-glycine units. There is a dominant IR vibration in the region of 1651–1658 cm^−1^ (6.0 µm, this calculation, Fig. 4) that consists mainly of the C=O stretch mode within two anti-parallel (beta sheet) chains of glycine, named the “amide I” band^17^. However, at 3896 cm^−1^ (2.5 µm) there is a vigorous vibration in the Gly_OH_ hydroxyl group. This strong movement of the rectus O^**…**^H group is a novel finding and may be related to this conformer absorbing UV light at 480 nm. The accuracy of these calculations is expected to be better than 5%.

A beta sheet protein backbone gives in general an IR absorption in the region of 6 µm and we show this in S1, Fig. 2 for the “core unit” compared to the first 10 amino acids in the most conserved of all proteins, subunit c of the ATP synthase: DIDTAAKFIG. Subunit C can have a variety of conformations being the rotor of the mitochondrial ATP synthase complex^21^, and a calcium regulated ion channel^22^.

### Experimental

In this section we present UV/visible absorption measurements on hemoglycin crystals that confirm the predicted 480 nm feature. By extension, the presence of this absorption is proof that there exists within hemoglycin one or several “R” chirality hydroxylated glycine residues with the C-terminus adjacent to an iron atom. A subsidiary absorption observed at 580 nm is consistent with a minor presence of either “S” chirality hydroxy glycine or of unmodified glycine in a position adjacent to Fe, within the hemoglycin structure.

The absorption measurements were all made on samples that showed the characteristic X-ray diffraction patterns of fiber or mesh structures seen in crystals of Acfer 086^14^, Allende (unpublished) and in the Sutter’s Mill sample of the present work (X-ray details in S2). In the carbonaceous chondrites of the present work organic material comprises at most a few percent while samples are initially in the milligram weight range, so the resulting crystals from solvent extraction are very small, of the order of 100microns and below. Beamline I24 at Diamond Light Source^23^ is capable of measuring UV/visible absorbance from 300 to 840 nm in samples at this small size that are mounted in readiness for X-ray diffraction.

**Figure 5:**
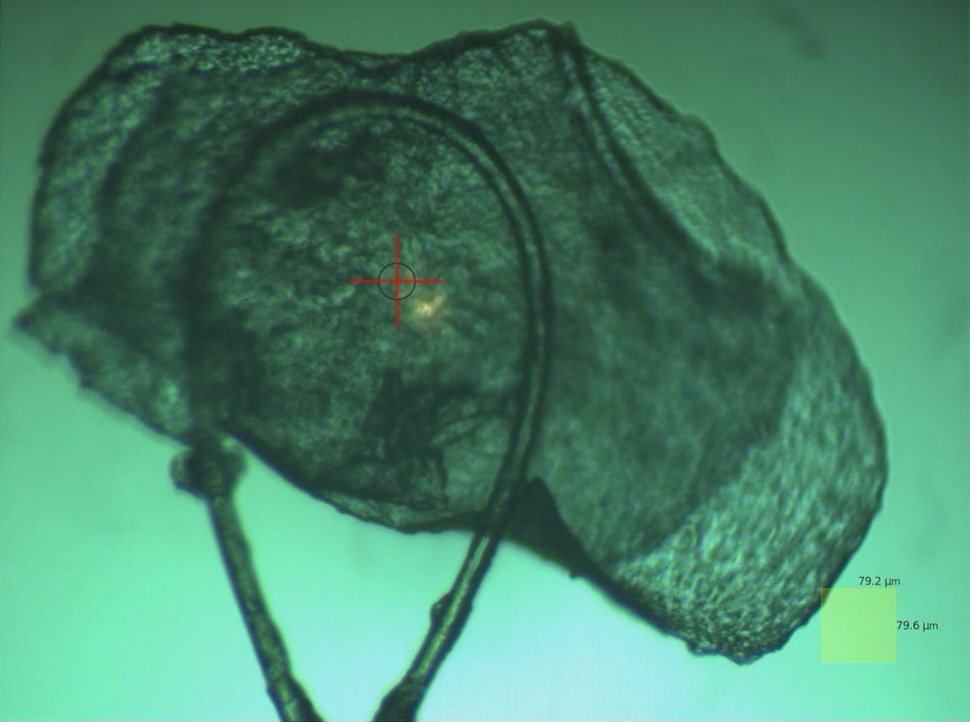
Sutter’s Mill crystal SM2 mounted in preparation for absorbance and diffraction measurements. Scale box at lower right 79.2 µm × 79.6 µm. Image taken by Diamond Light Source Beam line I24 by the beam line staff for JEMMc proposal MX31420.

A relatively thin, sheet-like crystal of Sutter’s Mill (Fig. 5) gave the most distinct 480 and 580 nm absorption data from the same region as the 50micron diameter X-ray target region.

**Figure 6:**
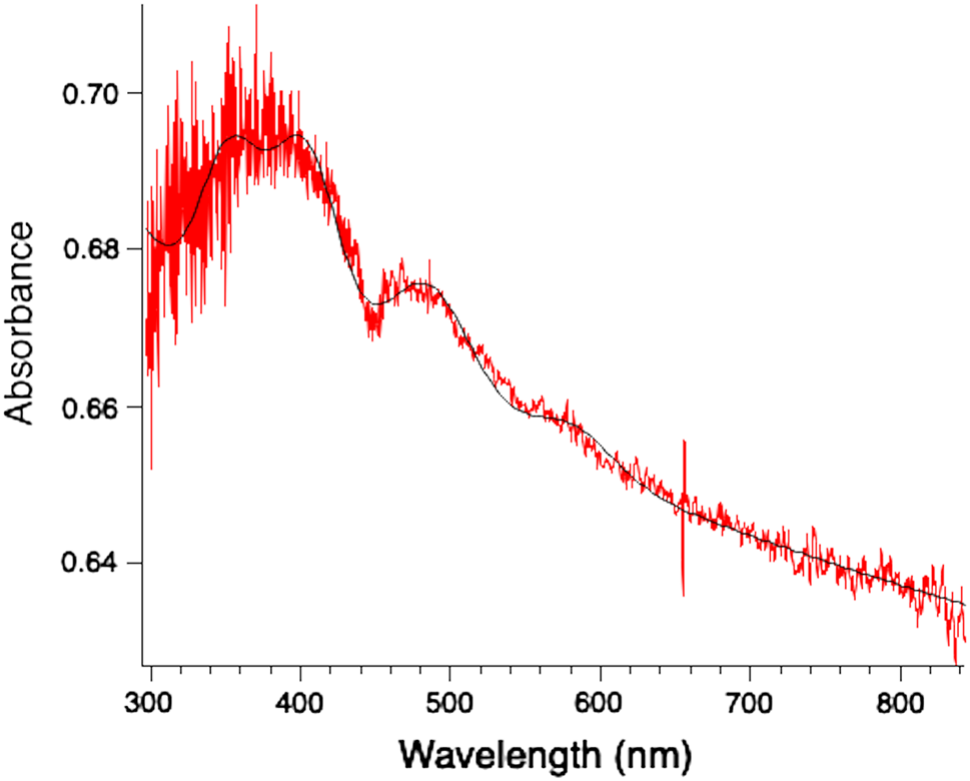
Absorbance of SM2 crystal, with fitted curve (black). The exposure time was 8 ms and number of accumulations 500. No data filter was applied. Curve fitting via Igor Pro 8 of Wavemetrics Inc.

An absorbance plot from this Sutter’s Mill crystal is shown in Fig. 6. The absorption contribution at 480 nm was evaluated by fitting a superposition of absorption features that corresponded to the main calculated features, with assumed Gaussian profiles as follows:

$$Absorbance = f(\lambda ) = ln\left[ {\frac{{a_{0} }}{{\lambda^{m} }} + \sum\limits_{J = 1}^{5} {a_{J} exp\left\{ {\frac{{(\lambda - \lambda_{J} )^{2} }}{{\Delta_{J}^{2} }}} \right\}} } \right]$$

where *a*_*J*_, * λ*_*J*_, and *Δ*_*J*_ are the amplitude, peak wavelength and half width at 1/e amplitude of a feature. The leading term $a_{0} /\lambda^{m}$ provides for both static system transmission and a minor amount of scattering that is coded by an index $m < < 1$. The template wavelengths from calculations, that are the starting point for least squares fitting, are listed in Table 3, along with final fitted values. Certain constraints were applied once values were established, so as to enable comparison of amplitudes. Widths were established as follows: *Δ*_1_ = *Δ*_2_ = *Δ*_3_ = 30 nm, and *Δ*_4_ = *Δ*_5_ = 37 nm. Although 480 nm was equally strong across the three samples, the 580 nm feature was relatively very low in the second and third samples, preventing an accurate fit.

**Table 3:** Fit coefficients for absorbance data.

Crystal	Run	Nominal wavelength before curve fit						
		*λ*_1_(nm)	*λ*_2_	*λ*_3_	*λ*_4_	*λ*_5_		
		330	350	390	480	580		

		*a*_*1*_	*a*_*2*_	*a*_*3*_	*a*_*4*_	*a*_*5*_	*a*_*0*_	*m*
SM2	1	0.01	0.043	0.046	0.0286 ± 0.001	0.0103 ± 0.0008	2.594	0.047
				Fit = **486 ± 0.09**				
APS 98512	2	0.01	0.046	0.240	0.0895 ± 0.0125	Not resolved	6.188	0.073
				Fit = **481.1 ± 2.5**				
SM2	2	0.01	0.095	0.206	0.122	Not resolved	7.530	0.102
				Fit = **481.4 ± 1.6**				
				Mean = **483 ± 3 nm**				

The best fits to the 480 nm feature gave an average center wavelength of 483 ± 3 nm (n = 3) in good agreement with the quantum calculation average of 477.5 nm. The SM2, run1 case allowed an estimate of the 480/580 nm absorption ratio = 2.8 ± 0.5 where each band was fitted with the same width for a true comparison. The computed RPA strengths of these bands are in the ratio 1.61, so at face value there are 1.74 times more absorbers from “R” chirality C-terminal hydroxy glycine at 480 nm than from the balance of “S” and “R” (S at either terminal and R at the N-terminal) at 580 nm. Purely random “S” and “R” formation would give an equal number. Two replication models in which the absorption of a 480 nm photon provides replication energy are discussed in S3. Each model predicts that there should be an “R” excess above “S” in the polymer in the steady state reached via many generations of replication.

## Discussion

It is tempting to speculate on the consequences of a chirality-driven absorption in the visible region when the molecule in question shows evidence, through its great dominance, of some form of replication^10,14^. We consider the mechanisms that could benefit from such a specific source of energy in the field of a new star. One of these is energy transfer into vibrations.

### Electronic to vibrational energy transfer

The electron promoted by 480 nm absorption has an available energy of 2.58 eV that may be expended in chemical activity or vibrational excitation before it has a chance to radiatively decay in an estimated 300 ns. If this electron moves down the molecular chain there will be energy transfer to collective vibrational modes. The electron wavelength $\lambda = h/p$ at 2.58 eV is 0.764 nm whereas the repeat length in the chain is *a* = 0.718 ± 0.004 nm, from the present calculations. When there is an exact match (resonance) between the electron wavelength and the period of the chain there is strong Bragg reflection of the electron and momentum transfer to the chain structure. A periodic potential along the chain, such as exists here, modifies the situation^24,25^ by creating propagating electron states just below the resonance energy, which is 2.92 eV for this chain, so our 2.58 eV electron can propagate while losing energy via scattering to collective vibrational modes. Without additional dissipation, as for instance when in the gas phase, this vibrational energy will re-distribute slowly within the whole molecule, with continuing localized surging according to the Fermi simulation^26^. Sufficient energy will be available to “unzip” a series of hydrogen bonds spanning the length of the molecule, further discussed below.

### Replication

**Figure 7:**
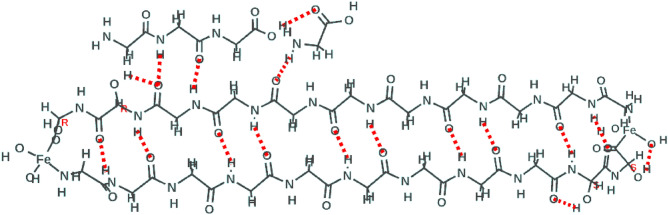
Cartoon of “core” replication process. Line molecular format. A peptide of 4 glycine units has started a replication and aligned to the core with a single glycine coming in to form an amide bond to make a 5-mer glycine. Hydrogen bonds in red. R/S labeling denotes chirality of hydroxy-glycine units. Spartan '20 Version 1.1.5 (220607) (Mac OS 12.5.1).

The core hemoglycin molecule is present as the largest sub-component in all the major mass spectrometry peaks in an extensive analysis^10^ of meteorite extracts, with support for its proposed structure in an X ray diffraction study^14^. It has been found to date in four different CV3 meteorites and its extreme dominance strongly suggests that it could have been formed in a process of molecular replication. Such a simple structure could in principle replicate as a template on which further glycine chains could condense while being held in the correct orientation by hydrogen bonds at the outer edge of the parent molecule (Fig. 7).

The length of the newly-condensed poly-glycine chain would be determined by the original, and if additional Fe atoms were present its ends could be capped, followed by the rapid completion of a new anti-parallel chain, which would be exothermic^27^. The new molecule would then need to break free. A thermodynamic argument quantifies the energy required for replication^28^. In the present case the energy should at a minimum be sufficient to break that line of hydrogen bonds, which in calculation amounts cumulatively to 71 kJ/mol^14^ (0.74 eV). In the models of replication considered in S3 there is additional energy input sufficient to disrupt at least one of the peptide-to-iron bonds. The most likely energy source is radiation from a new star into a young circumstellar disc.

Each of the two models presented in S3 assumes:

- Replication facilitated by the 480 nm absorption
- Equal “R” and “S” hydroxylation of available residues on daughter molecules
- Photon-limited absorption proportional to the number of 480 nm absorption sites (the ones at opposite ends of the molecule are independent from each other).

Model A assumes both N- and C-terminal hydroxylation, giving four sites in all, whereas model B assumes just two sites, one at each C-terminal of a polyglycine chain.

In the absence of a chiral absorption the models maintain a constant “R” = “S” proportion.

However, with the chiral 480 nm absorption each model predicts a long term excess of “R” over “S” with corresponding 480/580 nm absorption ratios 3.88 for model A and 3.37 for model B. The measured ratio is 2.8 ± 0.5. If the quantum chemical calculation is correct in its prediction of the factor of 1.61 in relative strengths at 480 and 580 nm, then this absorbance data indicates a chiral imbalance in favor of “R” over “S” in the samples, leading to the hypothesis that the chiral 480 nm absorption could be involved in hemoglycin’s replication. This caveat concerning the accuracy of the calculation is necessary because we are at the limit of knowledge concerning the precision of quantum chemistry in the treatment of a transition metal such as Fe in a relatively large organic molecule. The predicted wavelengths of absorptions, as had been anticipated before the experiments occurred, were found to be within a few tens of nm of the measured ones representing, in the visible region, an accuracy of about 5%. This gives assurance that the calculated set of electron wave functions should also deliver a good estimate of the transition strengths.

### Other implications for observational astronomy

There have been numerous reports of a 6 µm emission that is associated with certain forms of poly-aromatic hydrocarbons (PAH) (^29^, and references therein). This emission could also be due to a vibrational transition within the polymer amide backbone (Fig. 4, and Supplementary Figs. 1 and 2) where there is a dominant 6 µm line, shown here in absorption.

The Extended Red Emission (ERE) that is observed in circumstellar discs and the diffuse interstellar medium^30,31^ could have its origin in several of the many iron transitions in the red and near infrared region. The ERE phenomenon is characterized by a UV absorption, believed to be in the band 90 nm to 110 nm, followed by a red fluorescence^30^ that predominates between 540 and 760 nm. This type of fluorescence becomes possible in iron when parity forbidden transitions become allowed by chiral symmetry breaking in a certain molecular environment, as seen in the present work. A specific correlation of 6 µm emission with ERE would suggest an association of iron with polymer amide, either in the ISM or in a circumstellar cloud.

### Precursor role

Lei and Burton^32^ have described a route to genetic code development that begins with a polyglycine protocell. Following the cooling of Earth it is probable that there was significant in-fall of polymers such as hemoglycin which the present work shows to have been widely distributed in asteroids. Initially using a template mechanism, hemoglycin would have been able to replicate from available glycine and iron, forming a variety of lattices^14^ to fill volumes or cover surfaces.

## Conclusions

The hemoglycin molecule is found in four different meteorites and is a well-defined rod-like polymer of 22 glycine residues, several modified to hydroxyglycine, that also contains two iron atoms. Its constancy within all the most plentiful polymers analyzed by mass spectrometry leads to the strong probability that its constant, simple structure is propagated via template replication.

The 1494 Da core of hemoglycin space polymers was found in quantum chemical calculations to have a 480 nm absorption that depended specifically upon “R” chirality in the hydroxyglycine residue with its C terminus bonded to an iron atom. The default wavelength for this absorption, when “S” chiral, was calculated to be at 596 nm and 1.61 times weaker. Following these predictions, the absorption of hemoglycin crystals was measured to reveal for the first time a 483 ± 3 nm absorption feature and a much weaker 580 nm feature, these two being identified with the 480 and 596 nm predicted features. These findings gave solid support to the quantum calculation and showed that there is an “R” chirality hydroxyglycine adjacent to at least one of the Fe atoms in hemoglycin. Furthermore, the measured ratio of 483 nm/580 nm absorptions was higher than expected based on their calculated strengths and under the assumption that equal quantities of “R” and “S” chirality were present. Scenarios are explored in which hemoglycin undergoes template replication that depends upon absorption at 483 nm, with the consequence that “R” chirality is enhanced relative to “S” after several generations of replication. The measured 483 nm enhancement above 580 nm is consistent with one of these scenarios, pointing to the likely involvement of the 483 nm absorption in hemoglycin replication at the same time as posing a plausible replication mechanism.

## Materials and methods

### Theoretical

Modeling of this array of molecules was performed using Spartan ’20 software^33^, which incorporates a versatile and informative graphical user interface, executing calculations from the molecular modeling (MMFF) level up to advanced quantum calculations at the RPA level in embedded Q-chem^34^. Calculations were run on an M1 chip (Macbook Pro 17.1 with 8-core CPU used in 8 parallel threads). As the hemoglycin core unit had 780 active electrons in ab initio calculations at the 3-21G* level, it represented the practical upper limit for this computer and we were helped to run a longer glycine unit polymer calculation on Glycine_22_ Hydroxy-glycine_4_ Fe_2_O_4_ by Spartan Wavefunction chemists^33^ who could employ greater computing capacity for convergence. Absorption wavelengths and strengths were calculated to 20 excited states, which covered the range down to the mid-ultraviolet. Transition strengths were typically plotted on a log_10_ vertical scale unless mentioned otherwise, and an artificial width of 40 nm was applied to the spectral peaks to simulate the typical molecular broadening via vibrations. The calculations were all in gas phase, which we believe is more appropriate for molecules in molecular clouds or discs.

### Experimental

**Figure 8:**
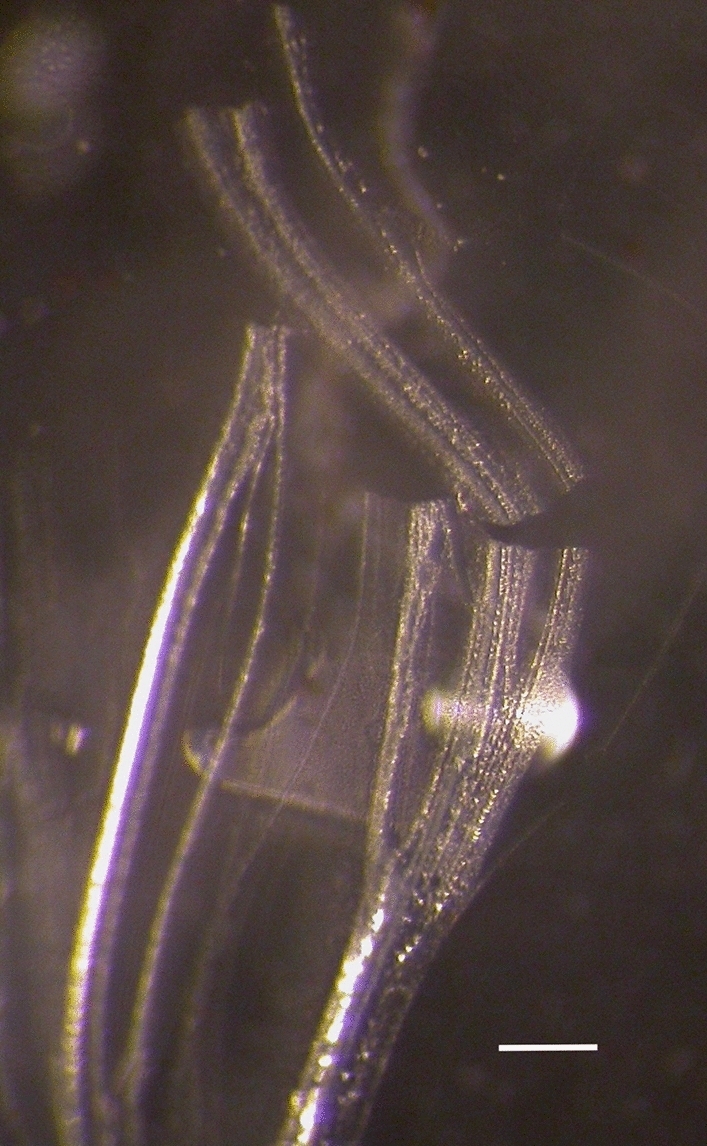
Sheets and tubes of hemoglycin in watch glass prior to crystallography loop pick-up. Scale bar 100 µm. Nikon E4500 camera mounted on a Leica MZ8 zoom Microscope.

The hemoglycin polymer is extracted from Acfer 086 and Sutter’s Mill meteorite samples. All procedures involving the meteorite samples are performed in a cleanroom to avoid terrestrial contamination. The Acfer 086 sample (from the Harvard Mineralogical and Geological Museum) was etched to form micron particles by methods already described^11,14^. The Sutter’s Mill sample was sent to us by NASA and the very small fragments were kept intact. For Acfer 086 and Sutter’s Mill samples the polymer was solvated by Folch extraction which involves placing 2 mg of micron particles or just a small fragment in a glass 2 ml or 1 ml V-vial and adding the solvents chloroform:methanol:water = 3.2:2:1 to produce a two-phase solvation system. The V-vials were then capped and left undisturbed for at least 6 months at room temperature. Glass borosilicate V-vials are used because the progress of crystallization can be viewed with an iPhone camera that has 10X magnification, without tipping the V-vial. Initially an interphase layer of soft structured tubes and vesicles is seen^10,14^. Residual mineral material sits at the bottom of the V-vial. After six months there has been slow evaporation even through the cap of the V-vial, leaving a concentrated solution. Using a pipette 200 µl is removed from towards the bottom of the V-vial and placed in a watch glass under 25X zoom light microscope. Immediately a swirl of sheets and tubules appears (Fig. 8) and sheets are lifted out on a Hampton crystallography loop attached to a base. The sheets are soft and slightly flexible with a high concentration of hydrogen bonds from the Hemoglycin glycine rods which allows them to adhere to the Hampton loop. Within minutes they dry and become stiff and from that point they are stable for weeks to years. Labelled cryo-caps are placed over the loop and the whole assembly taped to a flexible rubber computer mouse pad (this damps transit bumps), bubbled wrapped to prevent any movement, and boxed for shipping overnight to a synchrotron.

UV–Vis absorption and X-ray diffraction data were collected at beamline I24, Diamond Light Source^23^. In situ UV–Vis absorption spectra were collected at room temperature (18-23C), using mirror lenses mounted in an off-axis geometry and a deuterium halogen light source. The UV–Vis focal spot was ~ 50 μm in diameter. Spectra were recorded over the wavelength range of 300–840 nm using a Andor Shamrock 303 imaging spectrograph. Spectra were collected using exposure times of 8–25 ms with 400–500 accumulations per spectrum. X-ray diffraction data were collected using a defocused X-ray beam of 50 × 50 μm^2^, an exposure time of 20 ms and X-rays of energy 12.40 keV. Data were recorded using a Pilatus P3 6 M detector.

## Supplementary Information

Supplementary Information.

## Data Availability

The datasets used and/or analyzed during the current study are available from the corresponding author on reasonable request.

## References

[1] McGeoch, J. E. M. & McGeoch, M. W. Chiral 480nm absorption in the hemoglycin space polymer https://arxiv.org/abs/2203.06130 (2022). 10.1038/s41598-022-21043-4 PMC9519966 36171277

[2] Pasteur, L. The Asymmetry of Natural Occurring Compounds (two lectures given to the Chemical Society of Paris, 1860), translated by G. M. Richardson. In *The Foundations of Stereochemistry* (American Book Company, 1901).

[3] Chyba C, Sagan C (1992). Endogenous production, exogenous delivery and impact shock synthesis of organic molecules: An inventory for the origins of life. Nature.

[4] Engel MH, Macko SA (1997). Isotopic evidence for extraterrestrial non-racemic amino acids in the Murchison meteorite. Nature.

[5] Cronin JR, Pizzarello S (1997). Enantiomeric excesses in meteoritic amino acids. Science.

[6] Glavin DP, Dworkin JP (2009). Enrichment of the amino acid l-isovaline by aqueous alteration on CI and CM meteorite parent bodies. Proc. Natl. Acad. Sci..

[7] Kawasaki T, Hatase K, Fujii Y, Jo K, Soai K, Pizzarello S (2006). The distribution of chiral asymmetry in meteorites: An investigation using asymmetric autocatalytic chiral sensors. Geochim. et Cosmochim. Acta.

[8] Soai K, Shibata T, Morioka H, Choji K (1995). Asymmetric Autocatalysis and amplification of enantiomeric excess of a chiral molecule. Nature.

[9] Pizzarello S, Williams LB (2012). Ammonia in the early solar system: An account from carbonaceous meteorites. Astrophys. J..

[10] McGeoch, M. W., Dikler, S. & McGeoch, J. E. M. Meteoritic Proteins with Glycine, Iron and Lithium https://arxiv.org/abs/2102.10700 [physics.chem-ph] (2021).

[11] McGeoch JEM, McGeoch MW (2015). Polymer amide in the Allende and Murchison meteorites. Meteorit. Planet. Sci..

[12] McGeoch, J. E. M. & McGeoch, M. W. *A 4641Da polymer of amino acids in Acfer-086 and Allende meteorites* https://arxiv.org/pdf/1707.09080.pdf (2017).

[13] McGeoch, M. W., Dikler, S. & McGeoch J. E. M. Hemolithin: A Meteorite Protein containing Iron and Lithium”, arXiv:2002.11688 [astro-ph.EP] (2020).

[14] McGeoch JEM, McGeoch MW (2021). Structural organization of space polymers. Phys. Fluids.

[15] McGeoch, M. W., Šamoril, T., Zapotok, D. & McGeoch J. E. M. Polymer amide as a carrier of ^15^N in Allende and Acfer 086 meteorites. To be submitted.

[16] Lotz B (1974). Crystal structures of polyglycine I. J. Mol. Biol..

[17] Moore WH, Krimm S (1976). Vibrational analysis of peptides, polypeptides and proteins. I Polyglycine I. Biopolymers.

[18] Corliss C, Sugar J (1982). Energy Levels of Iron, Fe I through Fe XXVI. J. Phys. Chem. Reference Data.

[19] Basic Atomic Spectroscopy Data, Fe I. https://physics.nist.gov/PhysRefData/Handbook/Tables/irontable5_a.htm.

[20] Basic Atomic Spectroscopy Data, Fe II. https://physics.nist.gov/PhysRefData/Handbook/Tables/irontable6_a.htm.

[21] Dyer MR, Walker JE (1993). Sequences of members of the human gene family for the c subunit of mitochondrial ATP synthase. Biochem J..

[22] McGeoch JEM, Guidotti G (1997). A 0.1-700Hz current through a voltage-clamped pore: candidate protein for initiator of neural oscillations. Brain Res..

[23] Diamond Light Source beamline I24 specifications https://www.diamond.ac.uk/Instruments/Mx/I24.html.

[24] Takagaki Y, Ferry DK (1992). Electronic conductance of a two-dimensional electron gas in the presence of periodic potentials. Phys. Rev. B.

[25] Roy CL, Mahapatra PK (1982). Bloch electrons in finite crystals in the presence of a uniform electric field. Phys. Rev. B.

[26] Fermi, E., Pasta, J. & Ulam, S. Studies of non-linear problems. Document LA-1940 (Los Alamos National Laboratory, 1955).

[27] McGeoch JEM, McGeoch MW (2014). Polymer Amide as an Early Topology. PLoS ONE.

[28] England JL (2013). Statistical physics of self-replication. J. Chem. Phys..

[29] Beegle LW, Wdowiak TJ, Harrison JG (2001). Hydrogenation of polycyclic aromatic hydrocarbons as a factor affecting the cosmic 6.2 micron emission band. Spectrochim. Acta Part A.

[30] Witt AN, Lai TS-Y (2020). Extended red emission: Observational constraints for models. Astrophys. Space Sci..

[31] Gordon KD, Witt AN, Friedman BC (1998). Detection of extended red emission in the diffuse interstellar medium. Astrophys. J..

[32] Lei L, Burton ZF (2021). Evolution of the genetic code. Transcription.

[33] Spartan ’20 (incorporating Q-chem 5.1) (Wavefunction Inc.)

[34] Shao Y (2015). Advances in molecular quantum chemistry contained in the Q-Chem 4 program package. Mol. Phys..

