# Supplementary material for: Chiral 480 nm absorption in the hemoglycin space polymer: a possible link to replication
Source: Sci Rep. 2022 Sep 28;12:16198. doi: 10.1038/s41598-022-21043-4 (PMC9519966; doi:10.1038/s41598-022-21043-4)
Supplement: Supplementary file 1 — Supplementary Information. [file 41598_2022_21043_MOESM1_ESM.docx]

**Chiral 480nm absorption in the hemoglycin space polymer: a possible link to replication.**

**Julie E. M. McGeoch and Malcolm W. McGeoch**

**S1. Supplementary quantum calculations**

**
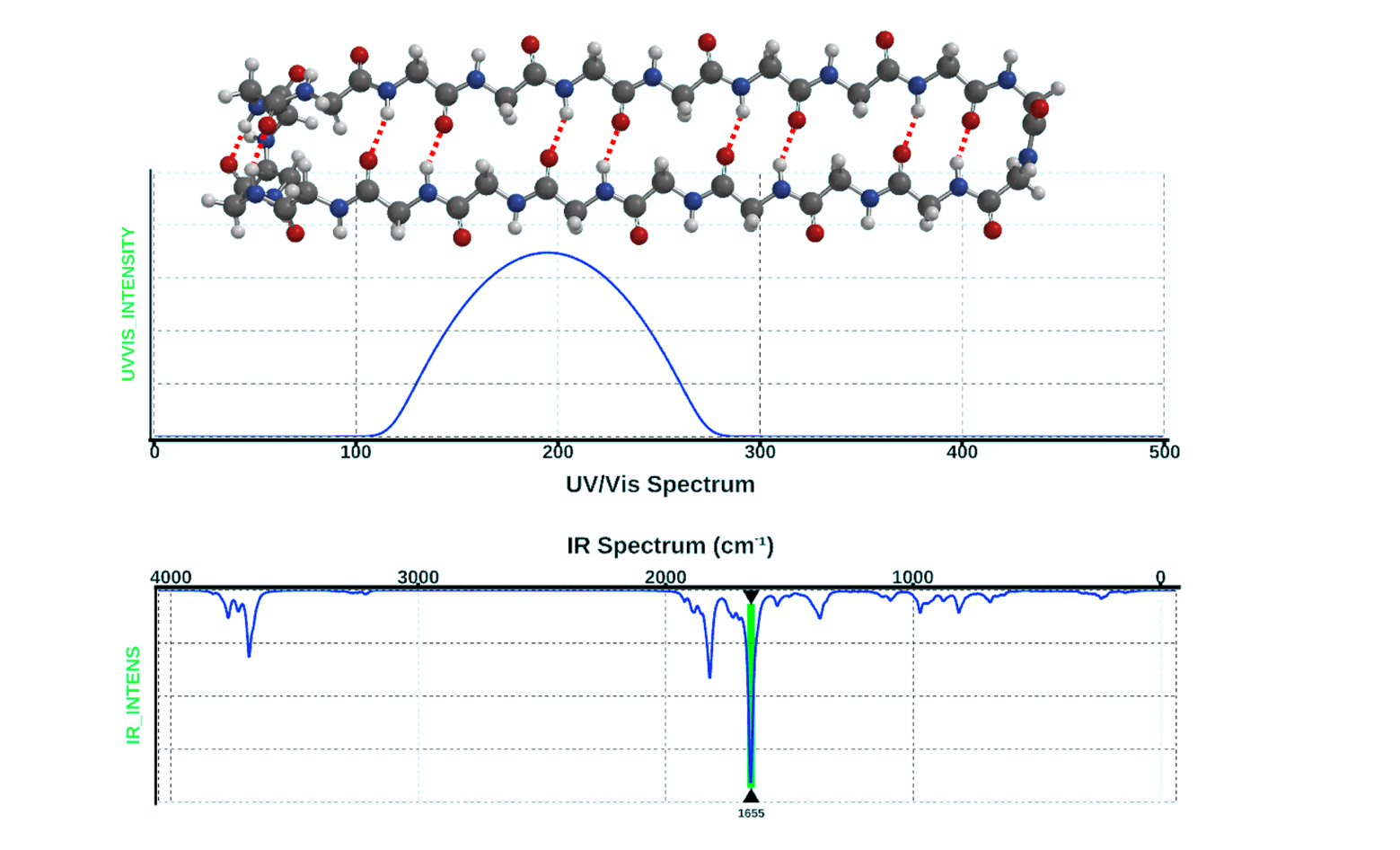
**

**Figure 1. Calculated** **UV/vis (nm) and IR (cm^-1^) absorptions from a loop of 22 glycine units. A loop with no Fe atoms typically absorbs from 150-200nm, shown here at 191.43nm. The IR high-lit peak at 1655cm^-1^ (6µm) is from the amide backbone. The molecular model format is ball and spoke. Atom labels: hydrogen white, carbon black, nitrogen blue, oxygen red.** Spartan '20 Version 1.1.5 (220607) (Mac OS 12.5.1).

**
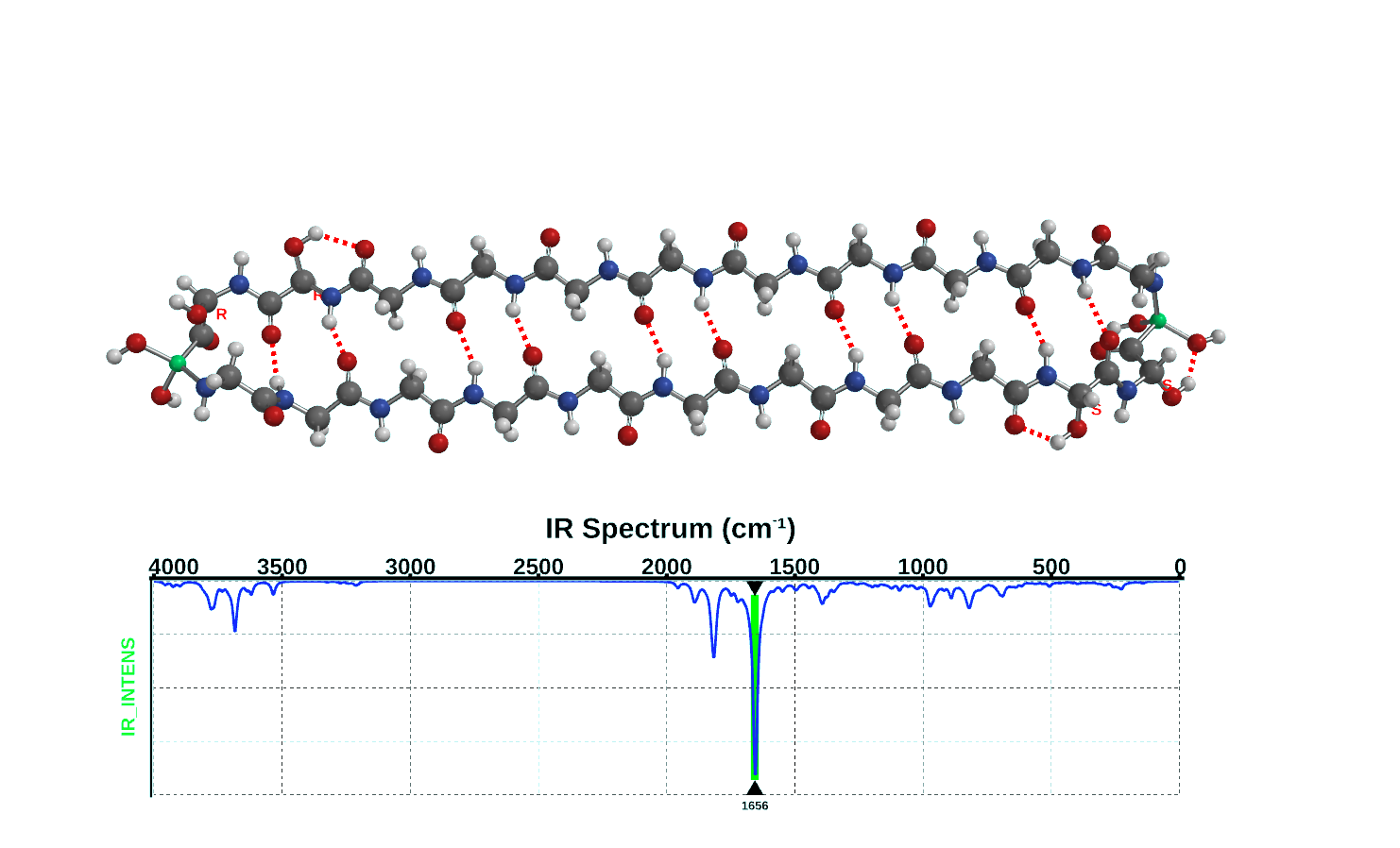
**

**
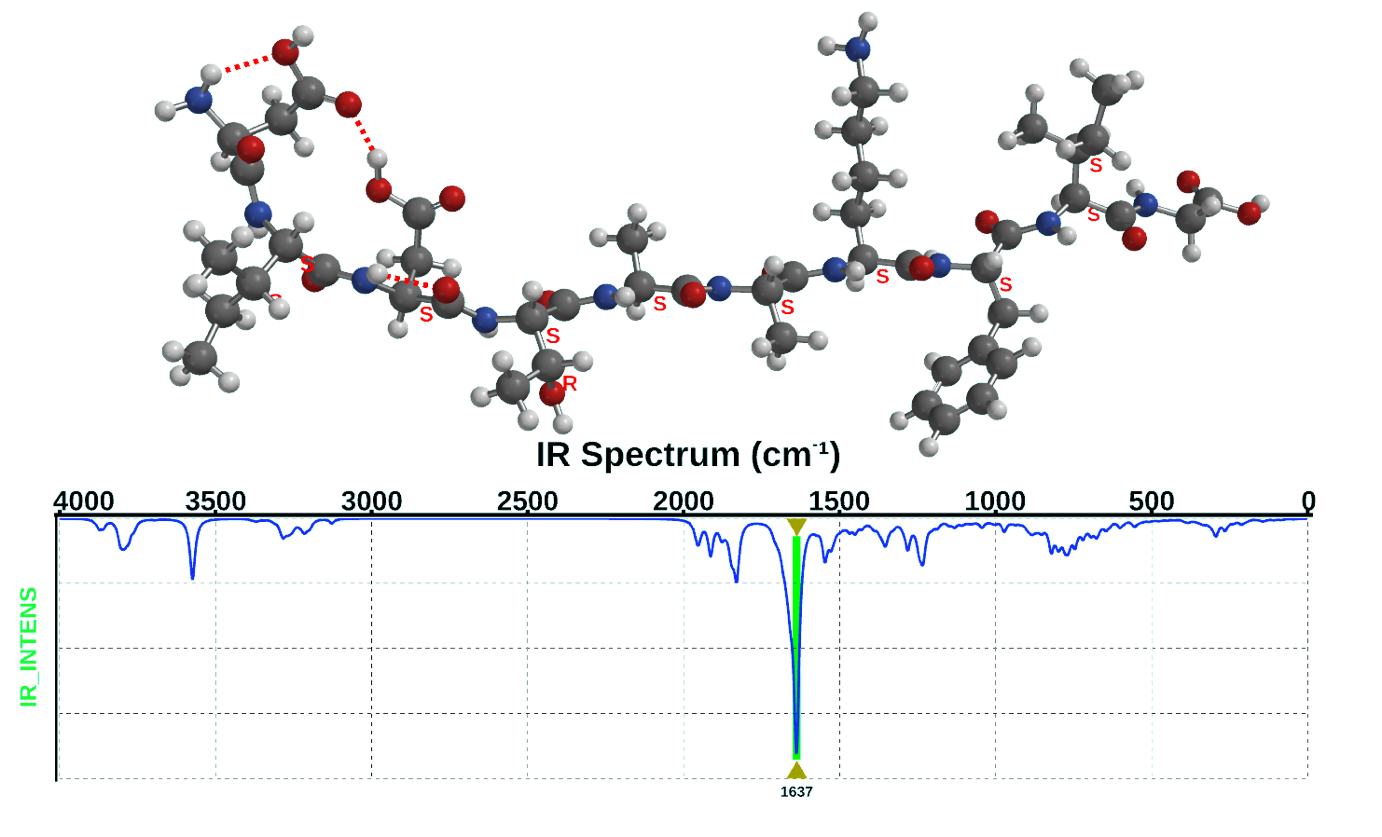
**

**Figure 2. A comparison of the IR absorption for the amide backbone of the “core” space polymer (above) with the 1st 10 amino acids of Subunit C of ATP synthase (DIDTAAKFIG) (below). The subunit C molecule is depicted as a beta sheet for an IR absorption comparison to the core space polymer. The amide backbone of both absorbs at 1635-1660cm-1 (region of 6µm). Molecule format is ball and spoke. Atom labels: hydrogen white, carbon black, nitrogen blue, oxygen red, iron green.** Spartan '20 Version 1.1.5 (220607) (Mac OS 12.5.1)

**S2. X-ray diffraction measurement**

Crystal SM2 (Sutter’s Mill) that yielded the clearest UV/visible absorption spectrum [SM2_spectrum1, Figure 6, main text] was the subject of an X-ray diffraction run under the following conditions:

Wavelength 1.000A

Distance 600mm

Angle step 0.1^O^

**
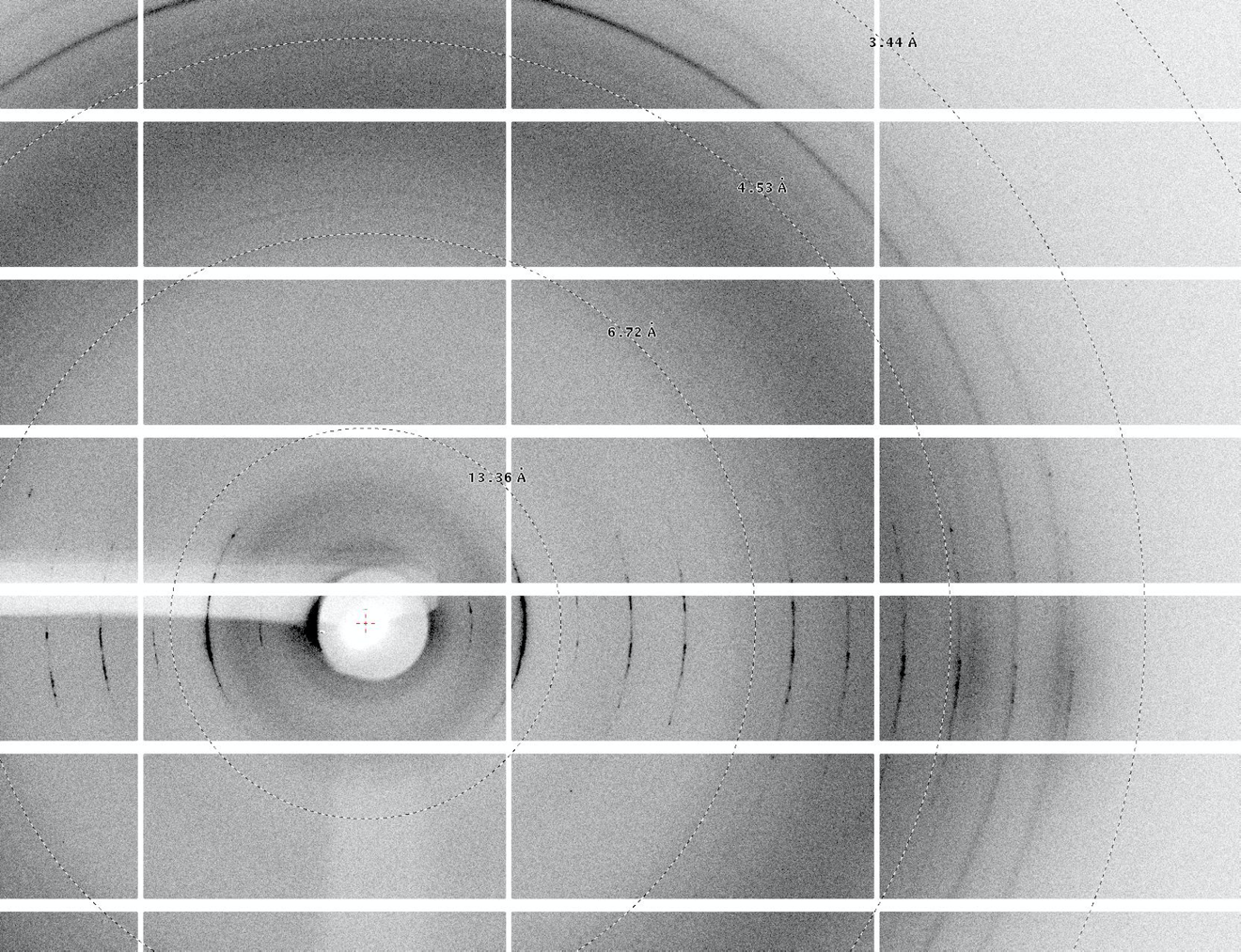
**

**Figure 3. Fiber diffraction pattern from crystal SM2**

Fiber diffraction was pronounced, with multiple orders seen, as for example in Figure 3. The fundamental order corresponded to a separation of 49.03 ± 0.18 Angstroms, which corresponded to the separation of lines of iron atoms within a square mesh of the core unit [14]. In rolled-up fibers of this mesh the spacing had earlier been determined in Acfer 086 to be 48.4 Angstroms [14] in agreement with the above result from crystal SM2. The crystals differed in that SM2 appeared to be in flat sheet form, whereas Acfer 086 (crystal APS 98512) was in rolled-up fiber form. The Acfer 086 crystal used in [14] was also studied for absorbance in the present work, data given in Table 3 of the main text.

The fitted diffraction orders for SM2 are listed in Table 1. Remarkably, orders 2 through 12 were resolved, with only 7^th^ order being absent. The 7^th^ order was more clearly visible when the images with ladder diffraction were summed (40 out of 3,600 images), however the fit was only applied to unsummed data without the 7^th^ order, for accuracy.

**Table 1. Fitting to diffraction orders of crystal SM2.**

| Order | Obs. (A) | Fit (A) |
| --- | --- | --- |
| 2 | 24.41 | 24.51 |
| 3 | 16.47 | 16.34 |
| 4 | 12.20 | 12.26 |
| 5 | 9.80 | 9.80 |
| 6 | 8.18 | 8.17 |
| 7 | - | - |
| 8 | 6.135 | 6.13 |
| 9 | 5.445 | 5.45 |
| 10 | 4.905 | 4.90 |
| 11 | 4.44 | 4.46 |
| 12 | 4.10 | 4.09 |

In summary, the identity of the SM2 crystal measured for absorbance was confirmed by observation of diffraction orders previously reported in Acfer 086 [14] which itself confirmed essential features of the “core unit” structure found by mass spectrometry [10].

**S3. Statistical Models of Replication**

The dominance of a single core molecular form of mass 1494Da within all the more intense m/z peaks in mass spectrometry [10] leads to the proposition that there is molecular replication, otherwise the dominance of a single length of polymer is hard to explain. We have X-ray confirmation of the main features of the 1494 structure [14], and in the main text of this paper there is now confirmation, via UV/visible absorption, of a specific feature of the molecule inferred from mass spectrometry, namely that it carries hydroxy-glycine residues adjacent to its terminal iron atoms. It was predicted in the first version of the main text [1] that there should be a 480nm absorption that depends specifically on having “R” chirality hydroxylation on the residue that has its C-terminus adjacent to a terminal iron atom, and this 480nm absorption has now been observed. When “S” chiral hydroxy glycine is at the same location the absorption is 1.61 times less strong and has a default wavelength of 580nm. The measured net absorption at 480nm relative to the default 580nm absorption is 2.8±0.5 in the Sutter’s Mill SM2 crystal where the latter can be clearly seen, whereas with a random equal distribution of “R” and “S” this ratio is calculated to be 1.61. If the absorption of 480nm light is necessary for replication, parent molecules with “R” type hydroxy glycine at the C-terminus next to iron will replicate faster. Over the course of many generations a bias toward “R” chirality will be established and reach equilibrium. Here two models of replication dependent upon 480nm absorption are explored to find the expected equilibrium “R” to “S” ratio. To anticipate the result of this section it is found computationally that a replication that is chirally dependent as described above, gives rise after a number of generations to a dominance of “R”/ “S” chirality within a sample in the ratio of 2.41 in model case “A” and 2.09 in model case “B”.

**Case A**

The molecule that we consider (slightly different from that in Figure 2, main text) has two hydroxylations, that can be “R” or “S” chirality, at each end of an anti-parallel glycine chain, specifically on the two residues adjacent to iron atoms at each end. We label the residue terminals “N” or “C” in the illustration in Figure 4, which shows a central core unit (the example is Type 9 from Table 1 in the main text) that has replication taking place from each side, with separation down the center of the parent molecule. To label the four hydroxyl groups a convention is adopted in which we begin at an N-terminal location and proceed via the adjacent Fe around the central core unit. The outer “daughter generation” units are labeled in the same convention. Given a central (parent molecule) set of chiralities, for example type 9 = SRRS, the outer possibilities under the same convention are (to the top) SRRS SRRR RRRS RRRR and to the bottom SSSS SSRS SRSS SRRS, as shown in the example of Figure 4. Altogether there are 16 cases of parent molecule to consider, listed in Table 2 and given numbers 1 to 16 for identification. Each of these gives rise to 8 daughter generation cases, four in each of the side units. The various products are summarized in the third column of Table 2 using their code numbers.

The final column of Table 2 gives the number of 480nm absorbing entities in a molecule, i.e. the number of C terminal R chirality hydroxyglycines, which is 0, 1 or 2. In the following calculation on the process of replication, we assume that the rate of replication is photon limited, and hence will be proportional to the number of absorbing entities in any given molecule. It seems likely that the density of a proto-planetary disc will be sufficiently high to create 480nm photon-limited conditions screened underneath a relatively thin outer UV-absorbing layer .


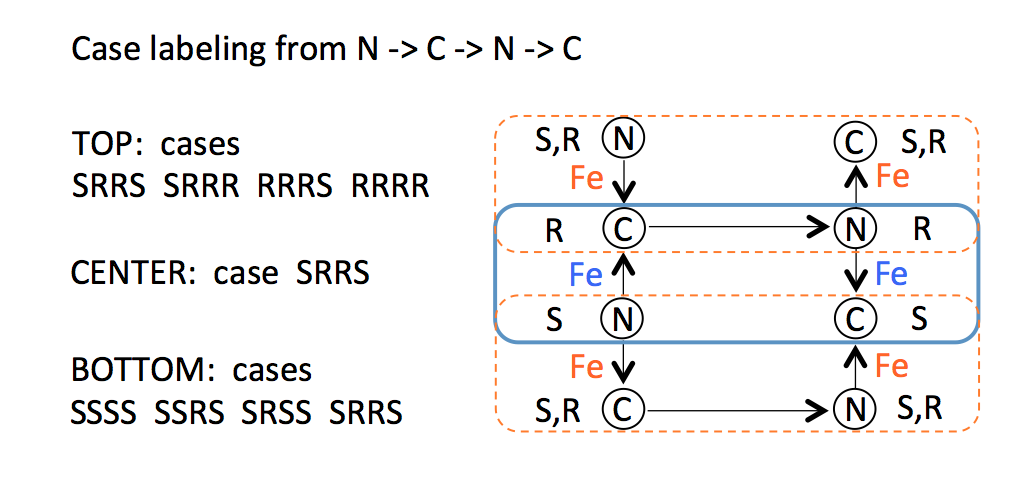


**Figure 4. Case labeling for a parent molecule in case 9 (SRRS, blue, center) undergoing two-sided replication to daughter molecules (orange) with random choice of daughter S,R chirality in newly formed hydroxy-glycine residues adjacent to iron (Fe).**

To summarize, it is proposed in case A that replication happens in four main steps:

1. Newly arrived unmodified glycine condenses into polymer glycine while becoming hydrogen-bonded in anti-parallel configuration to each side of a parent molecule (approach from one side shown in Figure 7 of the main text). There is evidence from mass spectrometry [10] for an intermediate m/z 2124 species that can be identified with a new 11-residue strand on one side.

2. If the complex carries one or more C-terminal “R” chirality hydroxy-glycines a 480nm photon is absorbed that provides the energy to split the line of hydrogen bonds down the center of the parent molecule, together with breaks between glycine and Fe, one at each end.

3. After the detachment of a daughter molecule there is attachment of two additional Fe atoms where needed.

4. An additional photon-driven reaction splits water to cause the hydroxylation of the new glycine residues adjacent to Fe. This is assumed for the purpose of these models to randomly create “S” or “R” chirality.

The above steps are followed in a program, for each of the 16 cases, through at least 10 generations in order to study the effect of replication via a chirally-dependent absorption. Convergence is rapid into the steady distribution listed in Table 3. Considering the “R” and “S” locations together with relative absorption rates and the factor of 1.61 in 480nm strength over 580nm, the case A absorption ratio 480nm/580nm in equilibrium is 3.88 times.

**Case B**

In contrast to case A, a simpler molecule is considered in which there are only two hydroxylations, each at the C-terminus of a glycine chain adjacent to iron.

These molecules can be coded by SS, SR, RS and RR, and again it is assumed that hydroxylation of the one open site on a daughter molecule is random between “R” and “S”.

Similarly, the process is taken to be 480nm photon limited, i.e. replication is twice as fast with configuration RR as RS or SR (SS cannot replicate at all).

**Table 2. Listing of cases and their products generated via random assignment of S, R chirality in the new hydroxy-glycine residues. Key in Figure 4.**

| Reference number | Case | Product cases | | | | | | | | Relative absorption rate at 480nm |
| --- | --- | --- | --- | --- | --- | --- | --- | --- | --- | --- |
| 1 | SSSS | 1 | 2 | 5 | 8 | 1 | 3 | 4 | 9 | 0 |
| 2 | SSSR | 1 | 2 | 5 | 8 | 2 | 10 | 11 | 12 | 1 |
| 3 | SSRS | 3 | 10 | 7 | 13 | 1 | 3 | 4 | 9 | 0 |
| 4 | SRSS | 4 | 11 | 6 | 14 | 1 | 3 | 4 | 9 | 1 |
| 5 | RSSS | 1 | 5 | 2 | 8 | 5 | 7 | 6 | 15 | 0 |
| 6 | RRSS | 4 | 11 | 6 | 14 | 5 | 7 | 6 | 15 | 1 |
| 7 | RSRS | 3 | 10 | 7 | 13 | 5 | 7 | 6 | 15 | 0 |
| 8 | RSSR | 1 | 2 | 5 | 8 | 8 | 13 | 14 | 16 | 1 |
| 9 | SRRS | 9 | 12 | 15 | 16 | 1 | 3 | 4 | 9 | 1 |
| 10 | SSRR | 3 | 10 | 7 | 13 | 2 | 10 | 11 | 12 | 1 |
| 11 | SRSR | 4 | 11 | 6 | 14 | 2 | 10 | 11 | 12 | 2 |
| 12 | SRRR | 9 | 12 | 15 | 16 | 2 | 10 | 11 | 12 | 2 |
| 13 | RSRR | 3 | 10 | 7 | 13 | 8 | 13 | 14 | 16 | 1 |
| 14 | RRSR | 4 | 11 | 6 | 14 | 8 | 13 | 14 | 16 | 2 |
| 15 | RRRS | 9 | 12 | 15 | 16 | 5 | 7 | 6 | 15 | 1 |
| 16 | RRRR | 9 | 12 | 15 | 16 | 8 | 13 | 14 | 16 | 2 |

The calculated case B equilibrium distribution is:

SS 8.15%; SR 22.77%; RS 25.63%; RR 43.45%.

Considering the relative absorption rates and the factor of 1.61 in 480nm strength over 580nm, the case B absorption ratio 480nm/580nm in equilibrium is 3.37 times.

**Summary**

Each of the two models assumes:

a) Replication facilitated by the 480nm absorption

b) Equal “R” and “S” hydroxylation of available residues on daughter molecules

c) Photon-limited absorption proportional to the number of 480nm absorption sites (the ones at opposite ends of the molecule are independent from each other).

Model A assumes both N- and C-terminal hydroxylation, giving four sites in all, whereas model B assumes just two sites, one at each C-terminal.

In the replication equilibrium each model predicts an excess of “R” over “S” with corresponding 480/580nm absorption ratios 3.88 for model A and 3.37 for model B. The measured ratio is 2.8±0.5.

**Table 3. Replication case “A” equilibrium steady distribution. Components defined in Figure 4.**

| Case | R,S components | Proportion relative to case 16 = RRRR | Percentage |
| --- | --- | --- | --- |
| 1 | SSSS | 7.29 | 1.36 |
| 2 | SSSR | 15.17 | 2.83 |
| 3 | SSRS | 8.69 | 1.62 |
| 4 | SRSS | 20.35 | 3.79 |
| 5 | RSSS | 11.22 | 2.09 |
| 6 | RRSS | 20.36 | 3.80 |
| 7 | RSRS | 13.60 | 2.53 |
| 8 | RSSR | 31.33 | 5.84 |
| 9 | SRRS | 32.55 | 6.07 |
| 10 | SSRR | 22.40 | 4.17 |
| 11 | SRSR | 40.02 | 7.46 |
| 12 | SRRR | 58.66 | 10.93 |
| 13 | RSRR | 37.33 | 6.96 |
| 14 | RRSR | 64.02 | 11.93 |
| 15 | RRRS | 53.42 | 9.96 |
| 16 | RRRR | 100.00 | 18.64 |
